# Pattern and levels of spending allocated to HIV prevention programs in low- and middle-income countries

**DOI:** 10.1186/1471-2458-12-221

**Published:** 2012-03-21

**Authors:** Peter Amico, Benjamin Gobet, Carlos Avila-Figueroa, Christian Aran, Paul De Lay

**Affiliations:** 1 The Heller School for Social Policy and Management, Brandeis University, Boston, MA, USA; 2 Joint United Nations Programme on HIV/AIDS (UNAIDS), 20 Avenue Apia, Geneva, Switzerland; 3 Abt Associates, 4550 Montgomery Ave, Suite 800 North, Bethesda, MD 20814, USA

## Abstract

**Background:**

AIDS continues to spread at an estimated 2.6 new million infections per year, making the prevention of HIV transmission a critical public health issue. The dramatic growth in global resources for AIDS has produced a steady scale-up in treatment and care that has not been equally matched by preventive services. This paper is a detailed analysis of how countries are choosing to spend these more limited prevention funds.

**Methods:**

We analyzed prevention spending in 69 low- and middle-income countries with a variety of epidemic types, using data from national domestic spending reports. Spending information was from public and international sources and was analyzed based on the National AIDS Spending Assessment (NASA) methods and classifications.

**Results:**

Overall, prevention received 21% of HIV resources compared to 53% of funding allocated to treatment and care. Prevention relies primarily on international donors, who accounted for 65% of all prevention resources and 93% of funding in low-income countries. For the subset of 53 countries that provided detailed spending information, we found that 60% of prevention resources were spent in five areas: communication for social and behavioral change (16%), voluntary counselling and testing (14%), prevention of mother-to-child transmission (13%), blood safety (10%) and condom programs (7%). Only 7% of funding was spent on most-at-risk populations and less than 1% on male circumcision. Spending patterns did not consistently reflect current evidence and the HIV specific transmission context of each country.

**Conclusions:**

Despite recognition of its importance, countries are not allocating resources in ways that are likely to achieve the greatest impact on prevention across all epidemic types. Within prevention spending itself, a greater share of resources need to be matched with interventions that approximate the specific needs and drivers of each country's epidemic.

## Background

In the third decade of the HIV epidemic, people are starting to adopt safer sexual behaviors, reflecting the impact of HIV prevention and awareness efforts [1]. There is evidence linking prevention strategies to reduction of incidence through behavioral change programs [2]. Also, there are reports of prevention program effectiveness and published estimates of the effectiveness of HIV prevention interventions in changing sexual behaviors [3,4]. However, some studies have shown that countries often do not allocate resources in ways that are likely to achieve the greatest impact; countries with similar epidemic types and prevalence rates distribute resources in radically different ways [5-7].

The United Nations Political Declaration on AIDS called to expand prevention coverage by diversifying approaches and intensify efforts to end new HIV infections and reaffirm that prevention of HIV must be the cornerstone of national, regional and international responses to the HIV epidemic [8]. Governments also committed to re-double HIV prevention efforts by taking all measures to implement comprehensive, evidence-based prevention approaches, taking into account local circumstances, ethics and cultural values [8]. Even with gains in access to treatment, the number of people newly infected continues to outpace those that are put on treatment [9,10]. Prevention remains the paramount challenge of the HIV epidemic and the modes of prevention are evolving with the results of the HPT 052 study [11,12]. Even with gains in access to treatment and a global decline in the number of new infections, there were an estimated 2.6 million people newly infected with HIV in 2010 [1].

As the AIDS epidemic continues to evolve, revealing geographic variations between and within regions, countries are increasingly working to formulate responses specific to their particular contexts [13]. With the continuing scale-up of the national and international AIDS response, it is increasingly important to accurately track the origin of funds and how they are spent at the national level. This includes looking at whether countries are obtaining the maximum benefit and achieving expected outcomes from their AIDS resources [14]. The mapping of resource flows can help decision-makers monitor the effectiveness of their national programs and improve resource mobilization in underfunded areas. At the global level, this allows the international community to evaluate the status of its response and its financial accountability.

## Methods

An analysis of reported HIV prevention expenditures from 69 low- and middle- income countries in 2008 was conducted, taking into account epidemic types, prevention spending categories and country income levels. A subset of 53 countries contained detailed information by spending category. All expenditures, by programmatic activity and HIV services, were cross-tabulated by source of financing and stratified by income level. Spending information from public and international sources was analyzed based on the National AIDS Spending Assessment (NASA) methods and classifications [15]. Total prevention spending was collected using United Nations General Assembly Special Session on HIV/AIDS (UNGASS) Indicator No. 1 reports [14]. This indicator reports on domestic and international spending on AIDS by country, broken down into spending subcategories.

NASA is a tool developed by UNAIDS to measure all the resources included in a country's national HIV response and is based on the national health accounts framework [16,17]. In addition to reporting on UNGASS progress, the NASA methodology has been used to support countries in planning and monitoring their HIV activities. NASA applies standard accounting methods to reconstruct all transactions in a given country, 'following the money' from the funding sources to agents and providers, and eventually to beneficiary populations. These data are collected from every institution and organization that intervenes financially in the national response to HIV. And they are collected by the country's NASA taskforce and entered into a Resource Tracking System (RTS).

Countries were classified by national income level. Economies were divided according to their Gross National Income (GNI) per capita for the data collection year used, calculated using the World Bank Atlas Methods and grouped into four categories: low-income (US$ 935 or less); lower middle-income (US$ 936 - $3,705); upper middle-income (US$ 3,706 - $11,455); and high income (US$ 11,456 or more) [18]. All prices were converted into constant 2008 dollars.

Finally, countries were grouped by type of HIV epidemic using UNAIDS and WHO classification criteria [19]. This classification identifies three epidemic types-low level, concentrated and generalized-based on the current state of the epidemic and prevalence levels in each country. Low-level epidemics are defined as having prevalence below 1% in the general population. Concentrated epidemics are not yet generalized, but have expanded to greater than five percent among any sub-population group and are largely confined to most-at-risk populations-commercial sex workers (CSW), men who have sex with men (MSM) and injecting drug users (IDU).

South Africa is notably absent, due to the fact that they did not report their HIV spending in 2008.

## Results

The 69 low- and middle-income countries with available data spent a total of US$ 5.1 billion on the AIDS response in 2008. Out of the 69 countries, there are 32 low-income countries, 26 lower middle-income countries and 11 upper middle-income countries. Prevention amounted to US$ 1.1 (+/- 22 million) billion of this total (21%); however, the majority of spending was focused on treatment and care (53%). Table 1*(Reported total and per capita prevention spending, proportion of international funding and overall HIV funding allocated to prevention by epidemic type for 69 countries, in order of per capita spending, 2008 (USD)*) shows the absolute and per capita amounts spent on prevention by epidemic type, the level of financing coming from international sources, the proportion of overall HIV resources that were allocated to prevention and countries' ranking with respect to per capita spending.

**Table 1:** Reported total and per capita prevention spending, proportion of international funding and overall HIV funding allocated to prevention by epidemic type for 69 countries, in order of per capita spending, 2008 (USD)

Country	% HIV spending allocated to prevention	Total prevention spending (USD thousands)	% of total prevention spending funded by international donors	Per capita prevention spending	Ranking by per capita prevention spending
**Low-level epidemics**					

Kyrgyzstan	63	5,547	88	1.01	17

Georgia	40	3,218	77	0.75	24

Cuba	12	5,543	25	0.48	35

Tajikistan	47	2,930	77	0.42	42

Azerbaijan	70	3,512	27	0.39	45

Iran (Islamic Republic of)	57	20,402	9	0.27	51

Lao People's Democratic Republic	31	1,571	100	0.25	55

Bolivia	40	2,178	60	0.22	56

Bangladesh	69	25,566	100	0.16	61

Morocco	37	4,685	71	0.14	62

Pakistan	68	9,709	24	0.06	65

Sri Lanka	66	1,030	100	0.05	66

Philippines	53	3,462	74	0.04	67

Egypt	35	2,601	50	0.03	68

Algeria	11	432	41	0.01	69

**Concentrated epidemics**					

Republic of Moldova	70	8,966	59	2.28	6

Honduras	59	14,420	56	1.86	9

Costa Rica	33	6,480	9	1.39	11

El Salvador	21	8,338	25	1.35	12

Cambodia	38	19,929	79	1.31	13

Belarus	66	12,365	14	1.25	14

Chile	23	20,321	2	1.19	15

Mali	30	11,987	91	0.98	18

Argentina	14	35,216	12	0.88	22

Thailand	22	45,287	21	0.71	25

Dominican Republic	23	5,450	73	0.52	33

Ukraine	23	22,808	63	0.48	36

Peru	34	14,135	73	0.47	37

Mexico	19	50,606	1	0.45	39

Colombia	20	20,788	0	0.45	40

Viet Nam	36	39,344	89	0.44	41

Malaysia	45	11,000	0	0.39	44

Armenia	42	1,100	77	0.36	47

Gambia	11	563	100	0.34	49

Myanmar	47	15,546	94	0.27	52

Panama	7	922	77	0.26	53

Madagascar	42	5,003	75	0.26	54

Brazil	7	41,759	3	0.21	57

Venezuela	8	5,662	1	0.20	58

Niger	23	2,822	96	0.20	59

Paraguay	13	1,164	51	0.18	60

Somalia	18	1,088	100	0.12	63

Indonesia	50	24,703	61	0.10	64

**Generalized epidemics**					

Botswana	9	29,766	66	15.37	1

Lesotho	12	9,869	67	4.84	2

Kenya	24	158,619	97	4.14	3

Rwanda	26	29,308	98	3.16	4

Gabon	38	4,542	8	3.11	5

Uganda	22	64,185	100	1.97	7

Mozambique	27	38,543	97	1.86	8

Malawi	19	20,598	98	1.42	10

Congo	35	4,118	100	1.12	16

Cote d'Ivoire	31	19,417	99	0.94	19

Togo	38	5,887	97	0.89	20

Burkina Faso	27	12,956	71	0.88	21

Guinea-Bissau	34	1,238	96	0.78	23

Eritrea	25	3,574	93	0.71	26

Central African Republic	15	3,031	92	0.70	27

Angola	38	12,215	16	0.68	28

Benin	28	5,745	81	0.65	29

Senegal	28	7,148	87	0.60	30

Cameroon	29	11,435	82	0.59	31

Burundi	22	5,736	81	0.54	32

Chad	38	5,323	100	0.49	34

Equatorial Guinea	12	329	100	0.47	38

Guinea	31	4,087	100	0.40	43

Nigeria	15	57,949	89	0.38	46

Ghana	22	8,307	83	0.35	48

Democratic Republic of the Congo	21	18,115	97	0.28	50

**Figure 1:**
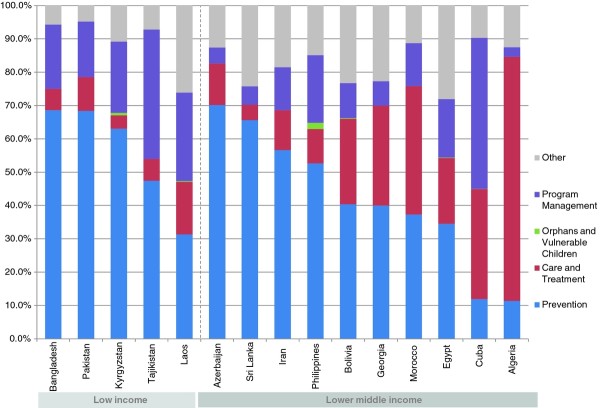
**Proportional distribution of AIDS spending in 15 low-level epidemics, by income level 2008 (USD)**.

**Figure 2:**
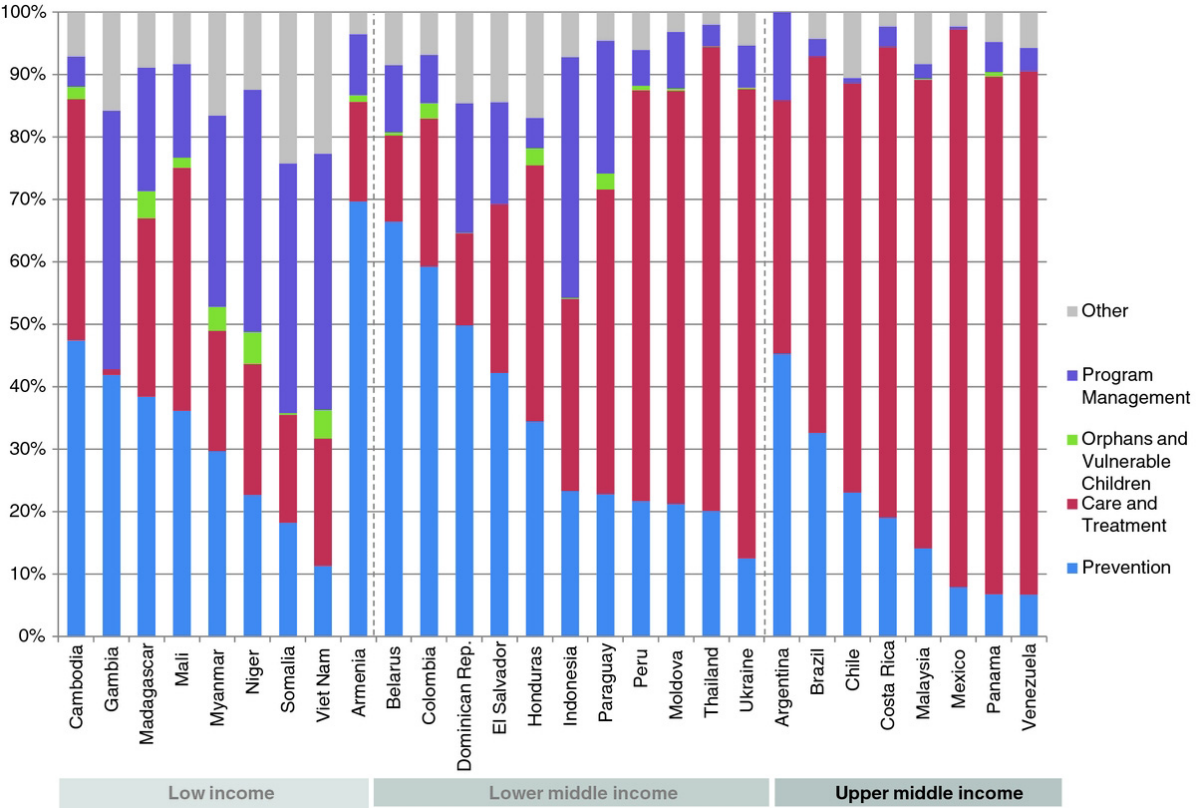
**Proportional distribution of AIDS spending in 28 concentrated epidemics, by income level 2008 (USD)**.

**Figure 3:**
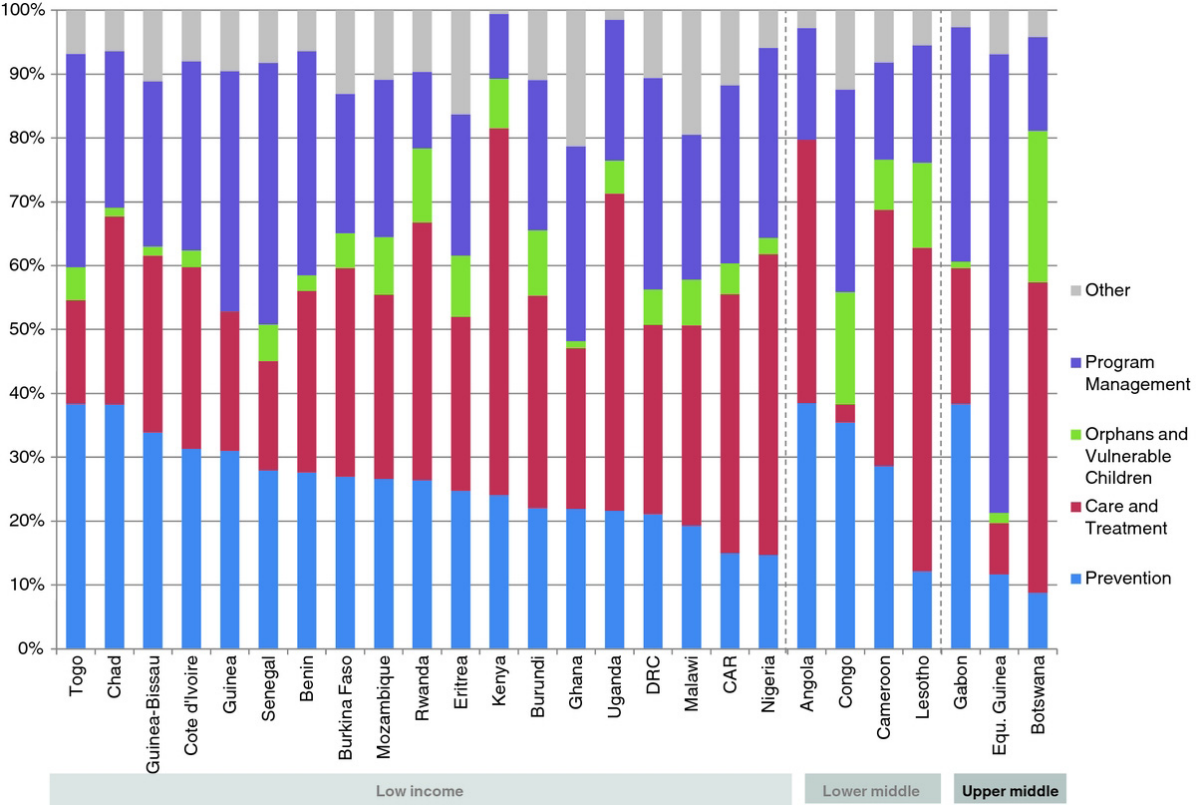
**Proportional distribution of AIDS spending in 26 generalized epidemics, by income level 2008 (USD)**.

The study includes 15 countries with low-level epidemics, 28 with concentrated epidemics and 26 with generalized epidemics. Once adjusted by the size of their populations, countries with generalized epidemics showed higher average per capita spending on prevention-US$ 1.82 (Range .28-15.37) compared to US$ 0.68 (Range .10-2.28) in concentrated epidemics and US$ 0.29 (Range .01-1.01) in low-level epidemics. The greatest share of HIV resources going to prevention was in low-level epidemics, where 45% of funds went to prevention, compared to 20% in concentrated epidemics and 21% for generalized epidemics. Figures 1, 2 and 3 provide a breakdown of HIV spending by epidemic type and income level for all countries. Prevention spending patterns vary greatly, even among similar epidemic profiles and income levels.

Currently, prevention relies heavily on international donors. On average 38% of total financing for prevention came from domestic-public sources, in contrast to the 63% of total treatment expenditures that were funded domestically. International funding for prevention was highest in low-income countries, where it reached a median value of 95% (Range 24-100%), compared to 59% (Range 0-100%) in lower-middle income countries and 8% (Range 0-100%) in upper-middle income ones. In 46 countries, international sources were responsible for over 60% of prevention resources, with contributions of greater than 80% in 31 of those countries. The majority of these are low-income countries, although this group also included Equatorial Guinea.

Many of the countries benefitting from international assistance were from sub-Saharan Africa, which registered both the highest levels of per capita spending on prevention and the greatest proportion of resources coming from international donors. Sub-Saharan Africa received 90% of its prevention resources from international donors, compared to 62% in South and South East Asia, 54% in Eastern Europe and Central Asia, 27% in the Middle East and North Africa, and 15% in Latin America and the Caribbean. Per capita, sub-Saharan African countries spent US$ 1.01 on prevention, the highest among any other region. Eastern Europe and Central Asia was the second highest spender at US$ 0.67 per capita, followed by Latin America and the Caribbean at US$ 0.43, South and South East Asia at US$ 0.21 and the Middle East and North Africa at US$ 0.12.

**Figure 4:**
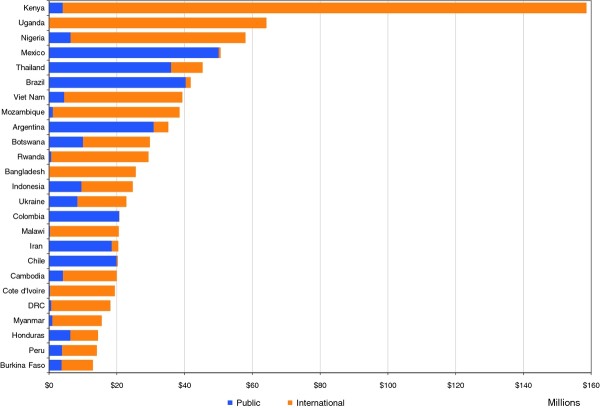
**Annual prevention spending from public and international sources, 25 top spending countries, 2008 (USD)**.

In absolute terms, Kenya (US$ 158. 6 million), Uganda (US$ 64.2 million), Nigeria (US$ 57.9 million), Mexico (US$ 50.6 million) and Thailand (US$ 45.3 million) are the biggest prevention spenders. Botswana (US$ 15.37), which has invested heavily in its AIDS programs in recent years, has the highest per capita spending, followed by Lesotho (US$ 4.84), Kenya (US$ 4.14), Rwanda (US$ 3.16) and Gabon (US$ 3.11). Figure 4 is a representation of the 25 countries that spent the most on prevention and it shows both domestic and public sources of finance. Among the top 25 spenders, 12 relied on international sources for over 75% of their expenditures.

Table 2 (*Total and proportional spending per prevention category in 53 countries, by epidemic type, 2008 (USD thousands)) *presents expenditure by type of epidemic in relation to 23 prevention categories for the subset of 53 countries that provided detailed reports. Communication for social and behavioral change (16%), voluntary counselling and testing (14%) and prevention of mother-to-child transmission (13%) received the largest share of funds. These were followed by blood safety (10%) and prevention and treatment of STIs (6%). Notably, male circumcision and post-exposure prophylaxis both received less than 1% of overall prevention funding. Condom-related categories including, social marketing, public and commercial sector male/female condom provision, accounted for 7% of funding.

**Table 2:** Total and proportional spending per prevention category in 53 countries, by epidemic type, 2008 (USD thousands)

			Low-level		Concentrated		Generalized	
AIDS spending category	**Total**	**% spending**	**Total**	**% spending**	**Total**	**% spending**	**Total**	**% spending**

Communication for social and behavioral change	107,373	16%	5,058	12	35,040	11	67,275	21

Voluntary counselling and testing (VCT)	91,408	14%	1,665	4	32,589	11	57,154	18

Prevention of mother-to-child transmission	87,913	13%	854	2	34,980	11	52,079	16

Blood safety	69,542	10%	5,252	13	41,271	13	23,019	7

Prevention, diagnosis and treatment of sexually transmitted infections (STI)	40,937	6%	1,507	4	36,081	12	3,348	1

Prevention activities not disaggregated by intervention	37,617	6%	287	1	5,576	2	31,755	10

Community mobilization	33,128	5%	1,194	3	19,259	6	12,675	4

Condom social marketing	30,441	5%	4,340	11	6,834	2	19,267	6

Risk-reduction for vulnerable and accessible populations	25,777	4%	2,725	7	18,650	6	4,402	1

Harm-reduction programmes for injecting drug users	22,244	3%	6,275	15	15,451	5	518	0

Prevention - Youth in school	21,414	3%	1,902	5	7,034	2	12,477	4

Prevention programmes for sex workers and their clients	19,764	3%	2,555	6	13,513	4	3,696	1

Public and commercial sector male condom provision	15,817	2%	554	1	4,153	1	11,110	3

Prevention programmes in the workplace	13,296	2%	474	1	3,657	1	9,165	3

Prevention activities not elsewhere classified	10,881	2%	1,439	4	8,102	3	1,340	0

Programmes for men who have sex with men	9,887	1%	1,691	4	7,989	3	207	0

Prevention - Youth out-of-school	9,426	1%	671	2	6,779	2	1,976	1

Safe medical injections	6,727	1%	1,425	3	16	0	5,287	2

Prevention of HIV transmission aimed at people living with HIV	5,254	1%	363	1	3,160	1	1,732	1

Universal precautions	4,802	1%	537	1	4,185	1	80	0

Post-exposure prophylaxis	2,647	0.00	1	0	2,451	1	194	0

Male circumcision	1,089	0.00	0	0	922	0	168	0

Public and commercial sector female condom provision	1,060	0.00	9	0	722	0	329	0

Microbicides	21	0.00	0	0	21	0	-	0

Programs for the most-at-risk-populations (MARPS) each received 3% or less of overall funding, although proportional spending was higher in low-level epidemics. Overall, low-level epidemics spent 25% of their prevention budget on higher risk groups, compared to 12% in concentrated epidemics and just over 1% in generalized epidemics. This was due mainly to higher investments in harm reduction programs, which received 15% of prevention funds in low-level epidemics, compared to 5% of total resources in concentrated epidemics and less than 1% in generalized epidemics. Programs for MSM were allocated 4% or less of resources in each epidemic type, while initiatives targeting CSWs received 6% or less.

## Discussion

The countries in this study spent just over a fifth of all resources for their AIDS response on prevention, providing a detailed picture of programmatic allocations of just over US$ 1 billion. While this analysis focused on prevention, these investments took place within the larger context of concurrent expenditures on treatment and care, orphans and vulnerable children, and program support and research, which brought total AIDS spending in 2008 to US$ 5.1 billion in the countries studied. Many of the prevention categories have low proportions of spending, but this does not necessarily mean that the spending is insufficient. This depends on the size of the target population and the amount that is spent. The recent Investment Framework proposes focused programs for high risk populations, elimination of HIV infections in children, reduction of risk through behaviour change, enhanced condom programs, treatment for people living with HIV and voluntary male circumcision in countries with high prevalence and low circumcision rates [20].

International funding is particularly prominent in prevention activities focused on MARPS, where it is the main source of overall funding, but it is likely that broader issues related to stigma, political will and human rights remain significant factors influencing domestic resource allocations. At least 42 countries in the study have laws criminalizing activities related to one or more MARPS [21]. Decreased donor contributions will result in reduced funding for these groups and domestic resources do not often make up the gap. Sustaining long-term preventive services in these populations could present a serious challenge, particularly in low-income countries.

Of the 26 countries with generalized epidemics in the study, 25 are located in sub-Saharan Africa, a region which accounts for an estimated two-thirds of the global HIV epidemic [22]. In these countries, it is essential to address sexual prevention, which have been reported as a key factor in the region's high levels of HIV transmission [9]. This requires simultaneous implementation of a variety of risk-reduction strategies. A key approach typically relies on messaging targeting a particular sub-population. In the 22 countries with generalized epidemics that provided a detailed breakdown of their spending, mass media campaigns, community mobilization and workplace prevention programs together accounted for 27% of prevention spending. VCT received 17%, while 20% was invested in PMTCT and 5% was put towards ensuring a safe blood supply. Communication for behavioral change was the top prevention spending category in generalized epidemics. Uganda has experienced success through its 1987 "Zero Grazing" campaign and appears to have reduced the percentage of men having multiple partnerships [23].

Currently, there is wide interest in using antiretroviral therapy as a means to prevent HIV transmission [24,25]. Evidence from PMTCT programs and follow-up studies of discordant couples has demonstrated a significant reduction in HIV transmission through ART [26]. Preliminary results from the HPTN 052 study show that ART is 96% effective in preventing transmission to an uninfected sexual partner in discordant couples where the index case has CD4 counts between 350 and 550 [11]. It is therefore plausible that early antiretroviral therapy and wide coverage could reduce community viral loads and significantly reduce the number of new cases of HIV [27,28]. HIV testing can act as an entry point to both effective prevention and treatment, and bridge the gap between these two approaches.

Increasing consistent use of condoms requires strategies that go beyond supplying condoms to increase demand and motivation for their use. Roughly, four percent of spending in generalized epidemics was allocated to the provision of condoms, while 3% of resources were used for condom social marketing activities. A few countries in the region did direct a large proportion of their prevention resources to condom-programs.

In generalized epidemics in sub-Saharan Africa male circumcision accounts for a small proportion of overall spending, with only four countries reporting expenditures in this area. Male circumcision has been shown to be highly cost effective [29,30]. A randomized control trial found that male circumcision has the potential to reduce the risk of HIV in men by 60% [29]. The lack of spending in this area may be understandable, given that wide advocacy for this option really began in 2008. In June 2009, Population Services International received a five-year, US $50 million grant from the Bill and Melinda Gates Foundation to provide voluntary male circumcision services to 650,000 men in Swaziland and Zambia, while Zimbabwe has expanded a pilot program and is now aiming to circumcise 80% of its male population by the end of 2025 [30,31]. There will likely be priority shifts and new trends in the future.

This study has several limitations; there is a lack of data to compare observed HIV spending levels with spending targets or populations at risk in the country. The data that do exist are highly unreliable and the authors determined that it was more instructive to present a global perspective of prevention than to compare spending to target population size. Also, expenditures are estimated using different sources of information and some countries lack comprehensive and regular expenditure records and accounting information systems. This analysis does not include out-of-pocket expenditures; although out-of-pocket spending has been found to vary from 23 to 68% of total health expenditures, the proportion that households divert to the purchase of condoms, HIV testing, clean syringes or other preventive interventions is unknown [32].

## Conclusions

Substantial changes are needed to achieve a more targeted and strategic approach to investment in the response to the HIV/AIDS epidemic that will yield long-term dividends. Until now, advocacy for resources has been done on the basis of a commodity approach that encouraged scaling up of numerous strategies in parallel, irrespective of their relative effects [20]. The United Nations Political Declaration on AIDS commits to ensure that financial resources for prevention are targeted to evidence-based prevention measures that reflect the specific nature of each country's epidemic by focusing on geographic locations, social networks and populations vulnerable to HIV infection [8]. It is important that prevention and treatment be viewed not as competing interests, but as complementary activities that together provide the basis for combined prevention approaches that address each country's context. This overview of prevention spending may be a catalyst for further research into a more strategic use of prevention investments.

## Competing interests

The authors declare that they have no competing interests.

## Authors' contributions

PA participated in the design, conception and analysis of the study and drafted the manuscript, BG participated in the design of the study and analysis, CAF participated in the design, conception and analysis of the study, CA participated in the analysis of the study and drafting of the manuscript, PD participated in the analysis of the study and provided detailed comments on the first draft. All authors read and approved the final manuscript.

## Pre-publication history

The pre-publication history for this paper can be accessed here:

http://www.biomedcentral.com/1471-2458/12/221/prepub
